# Bioprocess inspired formation of calcite mesocrystals by cation-mediated particle attachment mechanism

**DOI:** 10.1093/nsr/nwad014

**Published:** 2023-01-11

**Authors:** Qihang Wang, Bicheng Yuan, Wenyang Huang, Hang Ping, Jingjing Xie, Kun Wang, Weimin Wang, Zhaoyong Zou, Zhengyi Fu

**Affiliations:** State Key Laboratory of Advanced Technology for Materials Synthesis and Processing, Wuhan University of Technology, Wuhan 430070, China; Hubei Longzhong Laboratory, Xiangyang 441000, China; State Key Laboratory of Advanced Technology for Materials Synthesis and Processing, Wuhan University of Technology, Wuhan 430070, China; State Key Laboratory of Advanced Technology for Materials Synthesis and Processing, Wuhan University of Technology, Wuhan 430070, China; State Key Laboratory of Advanced Technology for Materials Synthesis and Processing, Wuhan University of Technology, Wuhan 430070, China; State Key Laboratory of Advanced Technology for Materials Synthesis and Processing, Wuhan University of Technology, Wuhan 430070, China; State Key Laboratory of Advanced Technology for Materials Synthesis and Processing, Wuhan University of Technology, Wuhan 430070, China; State Key Laboratory of Advanced Technology for Materials Synthesis and Processing, Wuhan University of Technology, Wuhan 430070, China; State Key Laboratory of Advanced Technology for Materials Synthesis and Processing, Wuhan University of Technology, Wuhan 430070, China; Hubei Longzhong Laboratory, Xiangyang 441000, China; State Key Laboratory of Advanced Technology for Materials Synthesis and Processing, Wuhan University of Technology, Wuhan 430070, China; Hubei Longzhong Laboratory, Xiangyang 441000, China

**Keywords:** nonclassical crystallization, mesocrystal, calcium carbonate, zinc ion, particle attachment

## Abstract

Calcite mesocrystals were proposed, and have been widely reported, to form in the presence of polymer additives via oriented assembly of nanoparticles. However, the formation mechanism and the role of polymer additives remain elusive. Here, inspired by the biomineralization process of sea urchin spine comprising magnesium calcite mesocrystals, we show that calcite mesocrystals could also be obtained via attachment of amorphous calcium carbonate (ACC) nanoparticles in the presence of inorganic zinc ions. Moreover, we demonstrate that zinc ions can induce the formation of temporarily stabilized amorphous nanoparticles of less than 20 nm at a significantly lower calcium carbonate concentration as compared to pure solution, which is energetically beneficial for the attachment and occlusion during calcite growth. The cation-mediated particle attachment crystallization significantly improves our understanding of mesocrystal formation mechanisms in biomineralization and offers new opportunities to bioprocess inspired inorganic ions regulated materials fabrication.

## Teaser

Zinc was used as a cationic additive to mediate the crystallization of amorphous 3 calcium carbonate, leading to the formation of calcite mesocrystals via a particle 4 attachment crystallization mechanism.

## INTRODUCTION

The process of crystal nucleation and growth has attracted massive attention and been extensively investigated as it is one of the most crucial parts of scientific fields such as chemistry, biology, material synthesis, etc. Since the middle of the last century, classical nucleation theory [1] was established to explain the pathways of crystal nucleation and growth, whereas, the monomer-by-monomer mechanism was not satisfactory in explaining numerous phenomena in biomineralization [2] or the morphology and texture of some synthetic crystals [3,4]. All these controversies have greatly promoted the development of nonclassical crystallization theories and the concept of crystallization by particle attachment has been proposed [5].

Calcium carbonate has been widely studied as a classical model in the past decades to understand crystallization mechanisms and biomineralization processes. Recent studies show that amorphous calcium carbonate (ACC) precursor plays crucial roles during the formation of crystalline biominerals with unique morphology and excellent mechanical properties [6–8]. Generally, the crystallization of additive-free ACC in solution is supposed mainly through a typical dissolution−recrystallization pathway [9,10]. However, during the formation of hard tissue in some invertebrates, like sea urchin spines [11,12], ACC nanoparticles first aggregate at the growth front and subsequently transform into magnesium calcite mesocrystals in which amorphous regions and macromolecules are embedded [13]. This delicate control of the biomineralization process is often ascribed to the presence of specific macromolecules, which are indeed important factors as demonstrated by numerous *in vitro* [14–17] and *in vivo* [18–20] studies. In addition, calcite mesocrystals have been widely reported to form in the presence of polymer additives via oriented assembly of nanoparticles. However, the formation mechanism of calcite mesocrystals and the role of polymer additives remain elusive.

Recent studies have shown that inorganic ions, such as Mg^2+^ [21–28] and PO_4_^3^^−^ [29,30], could also dramatically influence the crystallization pathways of calcium carbonate biominerals. In particular, calcite mesocrystals in sea urchin spine contain a significant amount of Mg^2+^, suggesting that Mg^2+^ might also play an important role during the formation of mesocrystals. Mg^2+^ can significantly improve the stability of ACC and control the polymorph selection of calcium carbonate [31–33]. Moreover, we have recently shown that a suitable concentration of Mg^2+^ can promote the crystallization of ACC into a new hydrated crystalline calcium carbonate phase, calcium carbonate hemihydrate (CaCO_3_·0.5H_2_O), whereas lower or higher concentration leads to the formation of anhydrous calcite or monohydrocalcite (CaCO_3_·H_2_O), respectively [34]. Possible reasons behind these unique abilities include smaller ionic radius and higher dehydration energy of Mg^2+^ as compared to those of Ca^2+^ [21,35], which would restrain the dehydration of ACC and regulate the nucleation and growth kinetics of anhydrous calcite [34]. These results lead us to think whether other divalent cations could influence the crystallization pathway of calcium carbonate and lead to the formation of calcite mesocrystals.

Here, inspired by the biomineralization process of sea urchin spine, which is formed via transformation of ACC in the presence of Mg^2+^, we utilize zinc ion (Zn^2+^) as a cationic additive to mediate the crystallization of ACC. Zn^2+^ is chosen because it shares some similarity with Mg^2+^, such as smaller ionic radius and lower hydration free energy when compared to Ca^2+^. It has also been reported that trace amounts of Zn^2+^ can slow down the nucleation rate of calcite, and strong adsorption of Zn^2+^ on calcite surface inhibits the growth rate of calcite [36]. We discover that Zn^2+^ could induce the formation of ACC with smaller particle size at a much lower carbonate concentration because Zn^2+^ has a stronger binding ability to carbonate than Ca^2+^ and Mg^2+^. More interestingly, calcite mesocrystals can be obtained by phase transformation of amorphous nanoparticles, where the crystal growth proceeds by attachment of spherical nanoparticles at specific step fronts. Our study demonstrates that particle attachment crystallization pathway can be promoted by inducing the formation of temporarily stabilized nanoparticles of less than 20 nm. Therefore, this work significantly improves our understanding of mesocrystal formation mechanisms and provides new insight for inorganic ions guided materials synthesis.

## RESULTS

**Figure 1.**
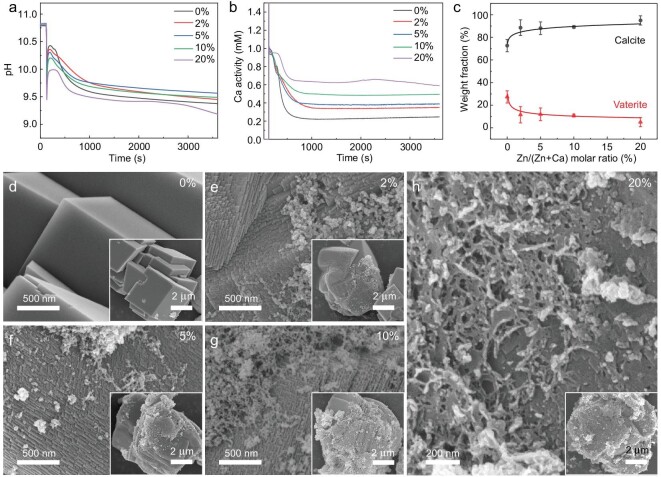
The evolution of pH (a) and Ca^2+^ ions activity (b) of the solution when varying amount of Zn^2+^ ions were present. (c) The weight fraction of calcite and vaterite in the refined phases of precipitates extracted at 3600 s. SEM images of the precipitates obtained via fast vacuum filtration at 3600 s when Zn/(Zn + Ca) was 0% (d), 2% (e), 5% (f), 10% (g) and 20% (h); the insets are corresponding low magnification images.

The crystallization pathway of ACC in the presence of Zn^2+^ was first investigated by adding pre-mixed ZnCl_2_ and CaCl_2_ solution into Na_2_CO_3_ solution, where the molar ratio of Zn^2+^ to total cationic ions (Ca^2+^ + Zn^2+^) ranges from 0% to 20% and the concentration of total cationic ions and Na_2_CO_3_ equals to 5 mM. The changes of pH and Ca^2+^ activity during reactions were monitored and recorded by pH meter and calcium ion selective electrode, respectively. Each experiment was repeated at least four times and results were summarized in Table S1. Fig. S1 shows that Zn^2+^ ions exhibit negligible effect on the measurement of calcium activity. According to the pH and Ca^2+^ activity curves (Fig. 1a and b), a significant drop of pH and an increase of Ca^2+^ activity indicate the precipitation of ACC immediately after the cationic solution was dosed into Na_2_CO_3_ solution at 120 s. The pH and calcium activity remained relative stable for a short period of time after the formation of ACC. The average pH at the plateau (160 s) decreased from 10.4 to 9.9 when the amount of Zn^2+^ increased from 0% to 20%, indicating that more CO_3_^2−^ ions were consumed during precipitation. However, the calcium activity after ACC formation remained relative constant for all reactions, suggesting that additional CO_3_^2−^ ions were consumed by Zn^2+^ ions and amorphous zinc carbonate formed.

Subsequent decrease in both pH and Ca^2+^ activity at ∼200 s for all reactions indicates the crystallization of ACC. It shows that the synthesized ACC was short-lived and Zn^2+^ less than 20% had little influence on the stability of ACC. In addition, comparing the pH and Ca^2+^ activity value after crystallization (from ∼1000 s to 3600 s), it can be found that the Ca^2+^ activity was higher for reaction with a higher initial amount of Zn^2+^ ions, while the pH first increased when the amount of Zn^2+^ increased from 0% to 5% and then decreased with further increasing of the amount of Zn^2+^. It suggests that either the solubility of crystalline calcium carbonate is higher with increasing initial amount of Zn^2+^, or the fraction of stabilized ACC increased. Moreover, for reaction with 20% Zn^2+^, a unique increase in calcium activity was observed at ∼2000 s, corresponding to the dissolution of calcium carbonate and release of Ca^2+^ and CO_3_^2−^ back to solution. However, the increase of Ca^2+^ activity was accompanied by the fast decrease of pH. These results indicate that crystallization of zinc carbonate phase occurred after the dissolution of amorphous phase.

The crystallization products at 3600 s were extracted from solution by vacuum filtration, followed by washing with ethanol and drying in a vacuum drying oven. According to the XRD patterns (Fig. S2), the precipitates were mainly a mixture of calcite and vaterite, however, characteristic peak of zinc carbonate hydroxide hydrate (ZCHH) at ∼33° was also observed when the initial amount of Zn^2+^ reached 20%. The average weight fraction of calcite and vaterite were calculated by Rietveld refinement of XRD patterns [37] (Fig. 1c) and ZCHH was not included during refinement. The average weight fraction of vaterite decreased from ∼27% to ∼5% when the initial amount of Zn^2+^ increased from 0% to 20%, demonstrating that the presence of Zn^2+^ can effectively inhibit the formation of vaterite. This behavior is quite similar to the effect of Mg^2+^ ions [34]. In addition, a clear shift of calcite diffraction peaks with increasing initial amount of Zn^2+^ was observed, indicating the incorporation of Zn^2+^ into calcite lattice (Fig. S2b). The average full width at half maximum (FWHM) of (104) peak of calcite was also calculated (Fig. S3) and the peak broadening as compared to control calcite suggests a reduced crystallite size and/or the existence of lattice strain. Accordingly, the crystallite size of the sample prepared in the presence of 5% Zn^2+^ was estimated to be ∼40 nm. Regarding the formation of ZCHH in the presence of 20% Zn^2+^, precipitates were extracted at around 1200 s, before the unique increase of calcium activity. XRD data show that no ZCHH was detected at this stage (Fig. S4). These results confirm that the increase of calcium activity at ∼2000 s was indeed caused by the transformation of amorphous phase to ZCHH.

The morphology of crystallized products was further investigated by scanning electron microscope (SEM). In the control group, calcite crystals exhibited typical rhombohedral shape with sharp edges and smooth surface (Fig. 1d). However, when Zn^2+^ was present (the amount of Zn ranged from 2% to 10%), the calcite crystals exhibited a morphology with rough surfaces and a significant number of growth fronts were arranged in a step pattern (Fig. 1e–g). Besides, significant amounts of spherical nanoparticles were observed attaching to the surface of calcite crystals, suggesting a particle attachment crystallization pathway. Interestingly, when the initial amount of Zn^2+^ reached 20%, the surfaces of calcite crystals were covered by thin sheet aggregates (Fig. 1h), presumably ZCHH crystals, which also appeared to be transformed from nanoparticles.

**Figure 2.**
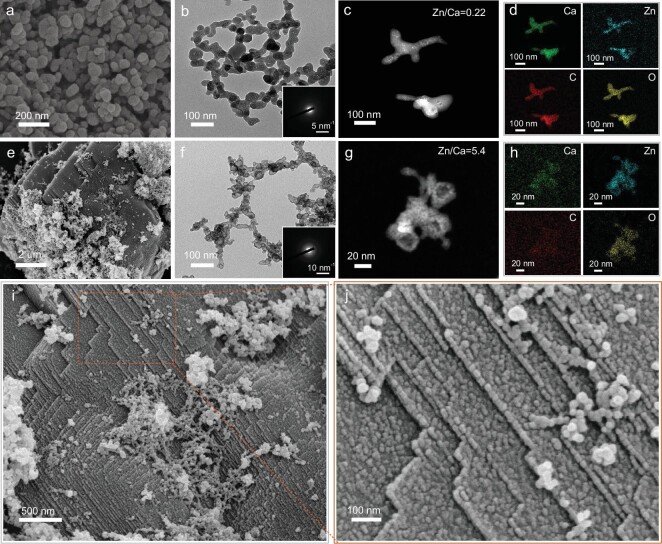
SEM (a), TEM (b), HAADF (c) images and EDX mapping (d) of Zn-ACC when Zn/(Zn + Ca) was 5%. (e) SEM image of calcite crystals at the early stage of crystallization when Zn/(Zn + Ca) was 5%. TEM (f), HAADF (g) images and EDX mapping (h) of nanoparticles extracted during crystallization. (i and j) Higher magnification SEM images of the crystal surface shown in (e). Insets in (b) and (f) are corresponding SAED patterns.

To investigate the transformation pathways from ACC to calcite in the presence of Zn^2+^, the precipitates were extracted from the reaction solution with 5% Zn/(Zn + Ca) at different time points. XRD pattern of the sample collected soon after ACC crystallization in solution at 400 s shows ∼90% of calcite and does not change significantly afterwards (Fig. S5). Because the polymorphic transformation from vaterite to calcite via a dissolu-tion–recrystallization process is relatively slow, this result indicates that initial calcite crystals were directly transformed from ACC without vaterite as an intermediate phase. SEM imaging (Fig. 2a) of ACC shows spherical nanoparticles with an average diameter of 51 ± 8 nm, which was significantly reduced as compared to pure ACC (∼200 nm, Fig. S6) [38]. Transmission electron microscope (TEM) imaging and corresponding selected area electron diffraction (SAED) pattern (Fig. 2b) confirm that the nanoparticles were amorphous. However, these nanoparticles were not stable under electron beam irradiation and bright spots could be observed in high angle annular dark field (HAADF) imaging (Fig. 2c). Energy dispersive X‐ray spectroscopy (EDX) mapping (Fig. 2d) shows that these bright spots are rich in Zn. The average Zn/Ca molar ratio of ACC nanoparticles is 0.22, which is much higher than the initial value of solution (0.05). It shows that Zn^2+^ has a stronger binding ability with carbonate as compared to Ca^2+^ and is favored to be incorporated in amorphous nanoparticles.

Figure. 2e illustrates the morphology of calcite crystals at the early stage of crystallization and many spherical nanoparticles were observed on the surface of calcite crystals. TEM images of nanoparticles extracted during crystallization show a hollow structure (Fig. 2f and g), which were still amorphous based on the SAED pattern. The shell of these hollow nanoparticles was composed of even smaller nanoparticles of less than 10 nm, in which Ca, Zn, C and O were uniformly distributed (Fig. 2h). The Zn/Ca molar ratio was 5.4, which was significantly higher than that of initial nanoparticles. These results suggest that initial amorphous nanoparticles experienced partial dissolution, in which ACC dissolved, while Zn-rich amorphous phase reprecipitated and accumulated on the surface of nanoparticles. Higher magnification SEM images (Fig. 2i and j) show that the surface of calcite crystal was clearly composed of nanoparticles of less than ∼20 nm. These nanoparticles were arranged in a fashion similar to the step-like growth front of calcite, confirming that the growth of calcite crystals proceeded via the attachment of nanoparticles. Besides these small building blocks, there were also some large nanoparticles lying at the step edges, suggesting that the smaller building blocks might be transformed from the initial nanoparticles.

**Figure 3.**
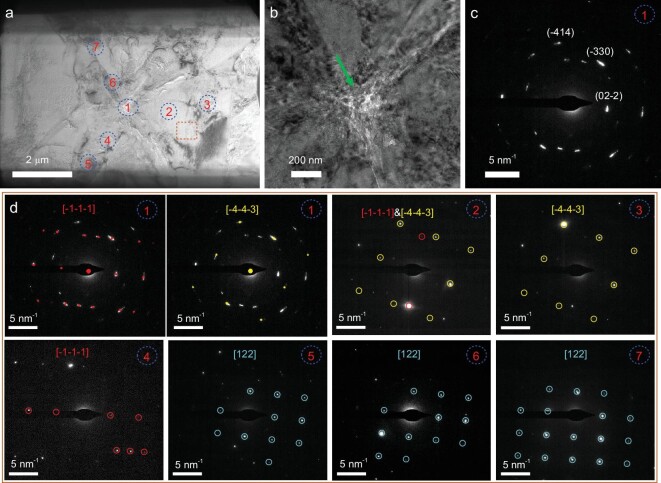
(a) TEM image of a thin lamella obtained from a calcite crystal that was extracted at the early stage of crystallization when Zn/(Zn + Ca) was 5%. The orange dotted square indicates the region for EDX mapping analysis in Fig. 4c and d. High resolution TEM image (b) and SAED pattern (c) of the central region of the crystal area 1 in (a). The green arrow in (b) indicates the presence of nanoparticles and pores. (d) SAED patterns in different areas marked in (a).

To further understand the crystallization process of calcite, a thin lamella of calcite crystal was obtained by focused ion beam (FIB) milling (Fig. S7). As shown in Fig. 3a, the central region (region 1) of calcite crystal is porous and composed of nanoparticles of ∼20 nm (pointed by green arrow, Fig. 3b). The corresponding SAED pattern (Fig. 3c) exhibits single crystal-like diffraction spots, however, the reflections have an angular spread of 10°, indicating that the nanocrystal domains are oriented over a 10° range. Furthermore, the pattern itself consists of two overlaid patterns, in which one crystal is observed along the [−1–1–1] lattice direction and the other one along the [−4–4–3] lattice direction and the angle between them is 5°. Simulated patterns define the reflections that belong to each crystal and these two crystals share the same crystal plane (−330). It confirms that the early stage of calcite formation proceeds by the aggregation of nanoparticles.

Moving from the central region of the crystal surface outwards, densely packed nanoparticles of ∼20 nm are observed (Fig. S8), indicating that the granular structures exist throughout the whole crystal. Electron diffraction patterns (Fig. 3d) of areas surrounding the central region were also collected. Moving from region 1 to regions 2 and 3, the relative intensity of the two crystals changes and the diffraction spots are more spherical without any arc shape, indicating that the crystals are more ordered. Moving from region 1 to regions 4 and 5, the orientation of crystal domains changes gradually from [−1–1–1] direction to [122] direction, while these two crystals share a common crystal plane (02–2). Similarly, regions 6 and 7 also belong to the crystal observed along the [122] direction. However, the orientation of crystal in region 7 is slightly different from the crystal in region 5, which is consistent with the misorientation of nanocrystal domains shown in region 1. Additionally, HRTEM images (Fig. S9) of a similar sample with nanoparticulate morphology show good lattice continuity and the corresponding FFT images are consistent with the overall electron diffraction patterns, confirming the single crystal structure. Altogether, it can be concluded that the obtained calcite crystals are mesocrystals. It should be noted that the size of nanocrystals observed from SEM and TEM images are smaller than the crystallite size estimated from the XRD pattern (∼40 nm), which could be due to the coalescence and fusion of some nanoparticles during crystallization.

**Figure 4.**
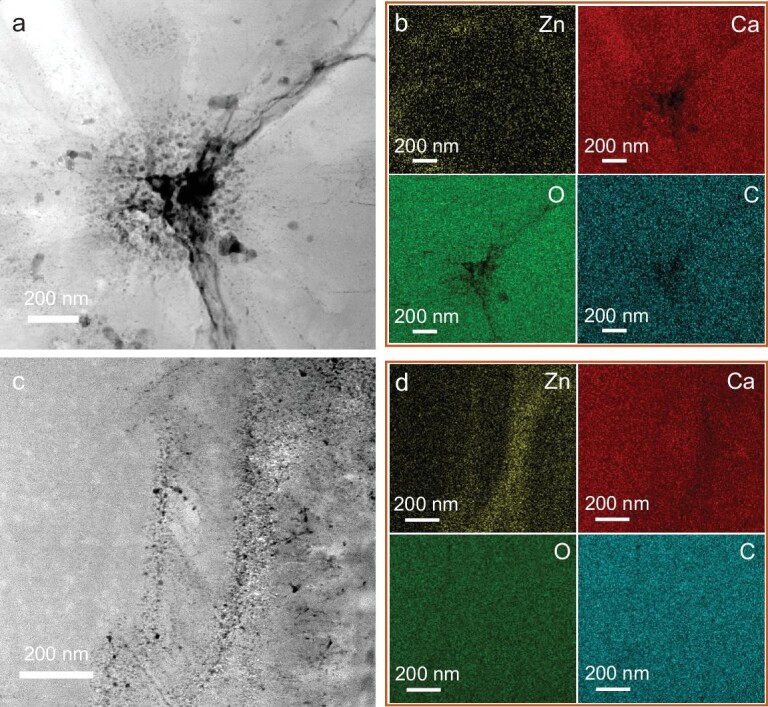
HAADF image (a) and EDX mapping (b) of the central region (region 1 in Fig. 3a). HAADF image (c) and EDX mapping (d) of the region that is framed by orange dotted square in Fig. 3a.

Besides, two regions (central region and the region framed by the orange dotted line in Fig. 3a) are selected for high resolution HAADF imaging and EDX mapping analysis. As shown in Fig. 4a, the central region consists of a significant number of nanopores. Furthermore, it is interesting that Zn^2+^ ions are dispersed ∼200 nm away from the central region, suggesting that during nucleation of calcite crystals, Zn^2+^ ions are squeezed out of the nanoparticles, however, they would still be trapped inside the crystals (Fig. 4b). In addition, many nanopores are also observed in a region away from the center (Fig. 4c), which are not uniformly distributed in calcite. Interestingly, regions with nanopores are often enriched in Zn^2+^ (Fig. 4d). This could be explained by the transformation of Zn-rich ACC nanoparticles with lower density to calcite with higher density after being occluded in the crystal, and Zn^2+^ is also likely to be squeezed out of the nanoparticles during crystallization. These results also suggest that the growth of calcite mesocrystals proceeds via attachment of amorphous nanoparticles.

## DISCUSSION

Nonclassical crystallization theory is widely used to explain the phenomena that are associated with biological mineralization, which is an important part in recent development of crystal nucleation and growth theory. It is well known that the formation of calcium carbonate mesocrystals in aqueous solution is often promoted by large organic molecules [39–42]. These organic molecules are usually rich in carboxylate groups that could have a strong binding with Ca^2+^ ions and induce the formation of stabilized amorphous nanoparticles. It has also been reported that high-magnesian calcite mesocrystals can be obtained by solid-state transformation of Mg-rich ACC, however, a high concentration of lipid was required to improve the stability of ACC nanoparticles [27]. Another strategy to form high-magnesian calcite mesocrystals is to perform the transformation of Mg-ACC in a nonaqueous environment, which precludes dissolution and reprecipitation during crystallization [43]. In this study, without using any organic molecules, we demonstrate that Zn^2+^ alone is able to induce the formation of calcite mesocrystals in aqueous solution by a particle attachment crystallization mechanism.

We propose that Zn^2+^ plays two major roles during this process. First, in stark contrast to the effect of Mg^2+^, a low concentration of Zn^2+^ is able to induce the formation of ACC nanoparticles with significantly reduced particle size, which would be beneficial for their aggregation and attachment to the crystal surface. The smaller particle size also reduces the energy barrier required to occlude these nanoparticles during crystal growth. However, the size of initial ACC nanoparticles (∼51 nm) is still too large to facilitate effective aggregation of nanoparticles. Zn^2+^ ions are also mostly incorporated in the amorphous nanoparticles due to a stronger binding with carbonate ions as compared to Ca^2+^ ions, and free Zn^2+^ ions in solution are not enough to effectively stabilize ACC nanoparticles. Therefore, initial ACC nanoparticles first undergo partial dissolution and form smaller (∼20 nm) and stable nanoparticles as the primary building blocks for calcite nucleation and growth, which could lower the free-energy barrier for particle aggregation and particle attachment crystallization. According to the pH and Ca activity curves (Fig. 1), the concentration of calcium carbonate after ACC formation was roughly 2 mM. To test whether Zn^2+^ is able to induce the formation of amorphous nanoparticles at such a low concentration, we performed direct mixing experiments at a total carbonate concentration of 2 mM with or without Zn^2+^. The results (Fig. S10) show that no immediate precipitation could be obtained for pure system, however, nanoparticles of ∼20 nm was obtained in the presence 5% Zn^2+^. It further confirms that Zn^2+^ could induce the phase separation of calcium carbonate solution and formation of amorphous nanoparticles at a significantly reduced calcium carbonate concentration.

**Figure 5.**
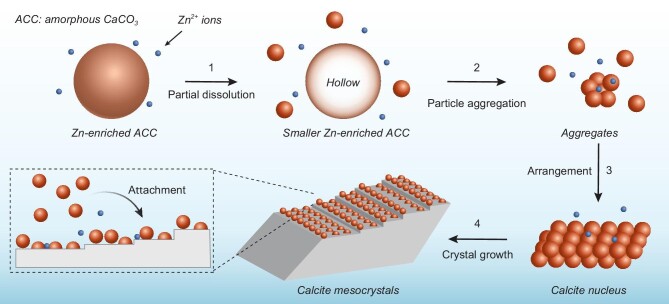
Schematic illustration of ACC crystallization pathways in the presence of zinc ions: (1) Zn-enriched ACC particles undergo partial dissolution and form Zn-enriched hollow amorphous particles. Simultaneously, smaller Zn-enriched amorphous particles are generated; (2) smaller Zn-enriched ACC nanoparticles form aggregates via particle aggregation; (3) nanoparticles in the aggregates arrange themselves and form the calcite nucleus; (4) calcite nucleus further grows via attachment of smaller Zn-enriched ACC nanoparticles and forms calcite mesocrystals.

Another role of Zn^2+^ during calcite crystallization is to inhibit the classical monomer-by-monomer growth pathway. Compared with Mg^2+^, Zn^2+^ has a smaller ionic radius and lower hydration free energy. The slow dehydration rate of Zn^2+^ makes it difficult to be incorporated in anhydrous calcite lattice. Previous studies have shown that trace amounts of Zn^2+^ can slow down the nucleation rate of calcite and strong adsorption of Zn^2+^ on calcite surface can inhibit the growth of calcite [36]. The strong bonding between Zn^2+^ and CO_3_^2−^ on the surface of calcite crystals makes it extremely difficult to remove Zn^2+^ adsorbed on calcite surface. In this situation, the attachment of smaller amorphous nanoparticles on the surface and subsequent occlusion within the crystal could be an alternative energetically favored crystallization pathway [44,45]. After the incorporation of amorphous nanoparticles inside calcite crystals, subsequent solid-state transformation leads to numerous pores together with Zn-enriched regions, as observed in the TEM images (Figs 3 and 4). Based on the above analysis, a schematic of the crystallization mechanism is illustrated in Fig. 5.

In this work, Zn^2+^ was selected as a representative divalent cation with small ionic radius and high dehydration energy. Besides Zn^2+^, other similar divalent cations like Co^2+^, Cu^2+^ and Fe^2+^ ions, etc., might also exhibit strong effects on the formation of ACC and crystallization pathways of calcium carbonate. For example, it has been reported that the presence of Co^2+^ in calcium carbonate solution promotes the formation of amorphous phases and, aragonite formation is favored at specific ratios of Co/Ca [46,47], which is consistent with our work. It should be noted that in some studies calcite precipitated in the presence of Co^2+^ exhibits a rod-shaped form, but is widely accepted to be a single crystal [42,48]. In these studies, the precipitation experiment was performed using the Kitano method, where crystallization of calcite is induced by slow removal of CO_2_ from Ca(HCO_3_)_2_ solution. However, our experiment was performed by directly adding a mixed solution of CaCl_2_ and ZnCl_2_ to Na_2_CO_3_ solution and amorphous nanoparticles precipitated immediately. Thus, crystallization by particle attachment is more likely in our experiment due to the presence of amorphous nanoparticles as the precursor.

Many studies have been reporting that Zn is an important essential trace element in multiple biological processes of living organisms. It is present in bone tissue, with preferential accumulation in the cement lines compared to the surrounding mineralized bone matrix [49]. Studies have also shown that zinc might play an important role in bone metabolism [50,51], though its presence and function have long been ignored assumedly due to the low occlusion level in biominerals. Our work suggests that zinc cations can have a potential impact on bone mineralization at relatively low concentrations, and most of them may be expelled after the formation of biominerals.

Various strategies have been used by organisms to strengthen the mineralized materials [13,52]. For example, the incorporation of amino acid, copolymer micelles and nanoparticles within crystalline lattices could increase the hardness and fracture toughness of the host crystals [53,54]. Recently, Polishchuk *et al*. reported that Mg-rich calcite nanoparticles segregate during or after transformation from amorphous to crystalline phase in the brittlestar lenses, where the coherent nanoprecipitates can induce compressive stresses on the host matrix and strengthen the biomineral [52]. In addition, Zn-substituted apatite was found in the incisor teeth of a prawn and a clear correlation between zinc substitution level and stiffness and hardness was observed [55]. Here, calcite mesocrystals also form via attachment of amorphous nanoparticles and Zn-rich regions segregate during crystallization. Therefore, it can be envisioned that the mechanical properties of the obtained calcite mesocrystals could be enhanced by similar mechanisms.

## CONCLUSION

To summarize, inspired by the formation process of magnesium calcite mesocrystals in sea urchin spine, we have investigated the crystallization pathways of ACC in the presence of Zn^2+^. The results indicate that Zn^2+^ can induce the formation of ACC nanoparticles at a significantly lower calcium carbonate concentration as compared to pure system and the particle size is significantly reduced. Subsequent crystallization of ACC leads to the formation of calcite mesocrystals, which proceeds via a particle attachment crystallization mechanism. The stronger bonding between Zn^2+^ and carbonate together with the slow dehydrate rate of hydrous Zn^2+^ are thought to be the key for promoting the attachment and occlusion of small amorphous nanoparticles. This work demonstrates that the formation of calcite mesocrystals can also be promoted by small amounts of cations, instead of commonly used organic macromolecules and provides new insight for inorganic ions guided materials synthesis.

## METHODS

### Materials and general preparative methods

Analytical grade calcium chloride dihydrate (CaCl_2_·2H_2_O), zinc chloride (ZnCl_2_) and sodium carbonate (Na_2_CO_3_) were purchased from Sigma-Aldrich without further purification. A mixed solution of 1 mol/L of ZnCl_2_ and CaCl_2_ was first prepared by dissolving corresponding chemicals in distilled water and the molar fraction of Zn/(Zn + Ca) ranged from 0% to 20%. All experiments were carried out at (25 ± 0.5)°C in a 100 mL water bath beaker under stirring. A 0.25 mL mixed solution was injected into 49.75 mL Na_2_CO_3_ solution to achieve 50 mL solution. The final concentration of CO_3_^2−^ in solution equals to the total concentration of Zn^2+^ and Ca^2+^. The injection was controlled by an automatic potentiometric titrator (907 Titrando, Metrohm Ltd) via dosing units at a rate of 10 mL/min to ensure rapid mixing of the two solutions. Precipitates generated from the mother solution were obtained by fast vacuum filtration followed by rinsing with ethanol. The precipitates were stored in a vacuum drying oven before further characterization.

### Monitoring pH and calcium concentration in solution

The evolution of pH and Ca^2+^ concentration was monitored by a pH electrode and a calcium-sensitive electrode (Ca-ISE, Metrohm Ltd), respectively. The pH electrode was calibrated daily with standard buffers (Metrohm Ltd). The calcium-sensitive electrode was calibrated before each test by titrating 0.1 mL, 1 M CaCl_2_ solution into 50 mL distilled water at a rate of 0.01 mL/min. In the meantime, the pH of the solution was maintained at 10 by dosing 0.1 M NaOH solution. The injection of cation solution was carried out 120 s after the start of pH and Ca concentration measurement.

### X-ray powder diffraction

A Bruker D8-Advance X-ray powder diffractometer with Cu Kα radiation (λ = 1.5406 Å) was used to record the X-ray powder diffraction (XRD) pattern of samples, with scattering angles (2θ) from 10° to 90° and a scan speed of 0.33°/s. Rietveld refinement was performed with Fullprof software [37]. Only calcite and vaterite were considered during refinement.

### Electron microscopy

SEM micrographs were collected using a JEOL JSM-7500F field emission scanning electron microscope (JEOL Ltd) working at 5 kV. TEM analyses were performed using JEM-1400Plus (JEOL Ltd) and Talos F200S electron microscope (Thermo Fisher Scientific, USA), working at 120 kV and 200 kV, respectively.

### Focused ion/electron dual beam system

The thin lamella of calcite was obtained by a Helios NanoLab G3 UC (Thermo Fisher Scientific, USA) focused ion/electron dual beam system.

## Supplementary Material

nwad014_Supplemental_File
