# Supplementary material for: Bioprocess inspired formation of calcite mesocrystals by cation-mediated particle attachment mechanism
Source: Natl Sci Rev. 2023 Jan 11;10(4):nwad014. doi: 10.1093/nsr/nwad014 (PMC10029847; doi:10.1093/nsr/nwad014)
Supplement: nwad014_Supplemental_File [file nwad014_supplemental_file.pdf]

Supporting Information

Bioprocess inspired formation of calcite mesocrystals by cation-mediated particle attachment mechanism

Qihang Wang[1,2,ǂ], Bicheng Yuan[1,ǂ], Wenyang Huang[1], Hang Ping[1], Jingjing Xie[1], Kun Wang[1], Weimin Wang[1], Zhaoyong Zou[1,2,*] and Zhengyi Fu[1,2,*]

Table S1. Reproducibility of pH and Ca activity measurements. Each experiment was repeated at least four times.

Initial Zn/(Zn+Ca) molar ratio	pH at 110 s	pH at 160 s	pH at 3600 s	Ca activity at 160 s (mM)	Ca activity at 3600 s (mM)
0%	10.81±0.02	10.43±0.04	9.34±0.03	0.94±0.02	0.25±0.03
2%	10.78±0.02	10.31±0.02	9.49±0.08	0.97±0.02	0.35±0.01
5%	10.78±0.03	10.24±0.03	9.50±0.09	0.97±0.04	0.40±0.03
10%	10.82±0.02	10.14±0.02	9.47±0.03	0.96±0.01	0.49±0.05
20%	10.83±0.00	9.91±0.03	9.09±0.11	0.98±0.02	0.59±0.04

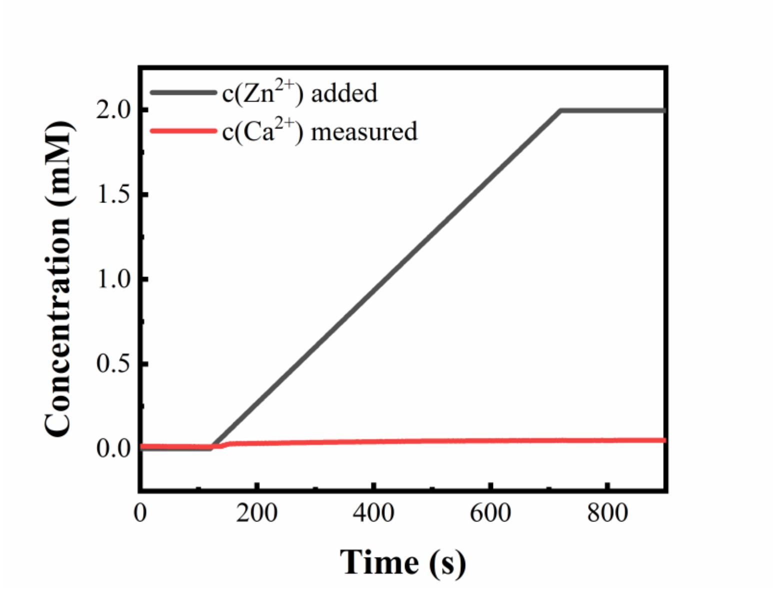

Figure S1. The influence of Zn^{2+} on the measurement of Ca^{2+} by calcium ISE. The measured concentration of Ca^{2+} by calcium ISE during continuous addition of $ZnCl_2$. It shows that the measured concentration of Ca^{2+} was always below 0.05 mM during continuous addition of Zn^{2+} up to 2 mM.

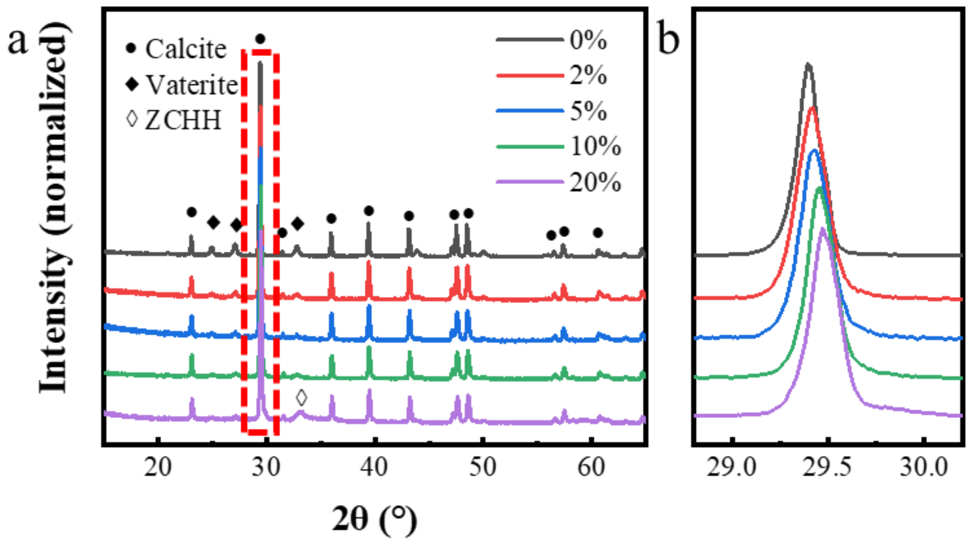

Figure S2. XRD patterns (a) of the precipitates extracted at 3600 s when Zn/(Zn+Ca) was 0%, 2%, 5%, 10% and 20%, respectively. (b) Magnified region of the (104) peak marked by dashed rectangle in (a). The peak shifts indicate the incorporation of Zn^{2+} into calcite lattice.

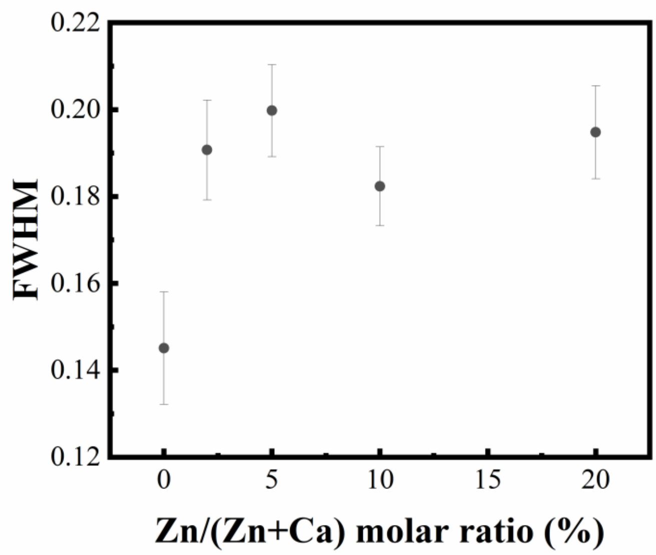

Figure S3. Average full width at half maximum (FWHM) of the (104) peak from XRD patterns of samples prepared in the presence of varying amount of Zn^{2+}.

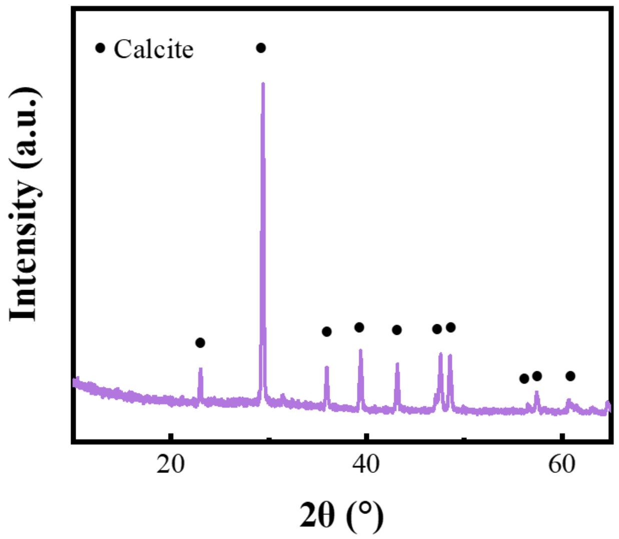

Figure S4. XRD pattern of the precipitates extracted at 1200 s when Zn/(Zn+Ca) was 20%. It indicates that the precipitates were calcite and no ZCHH was detected.

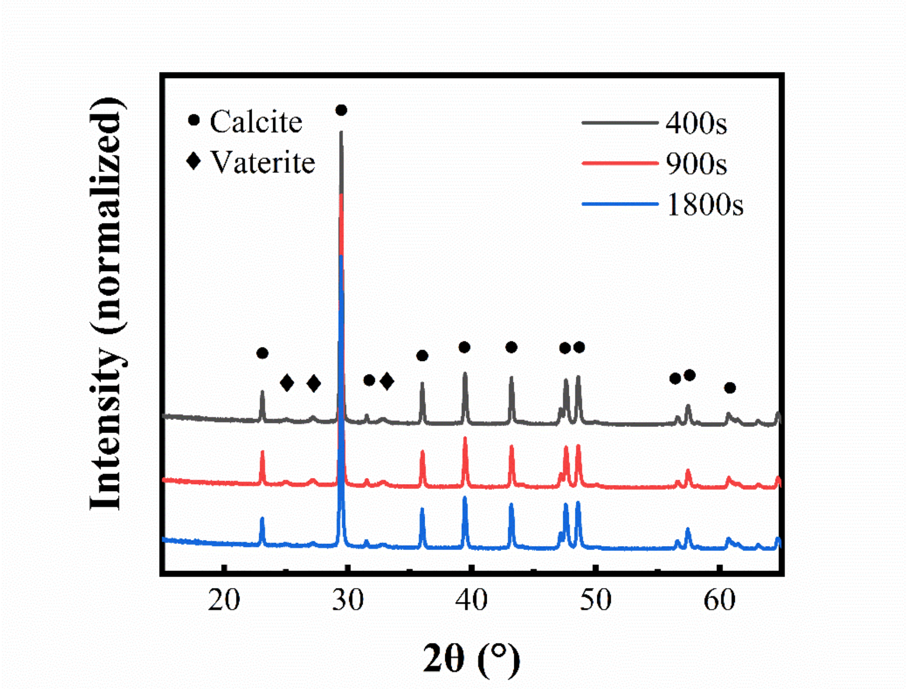

Figure S5. XRD patterns (a) of the precipitates extracted at different time points when initial Zn/(Zn+Ca) molar ratio was 5%.

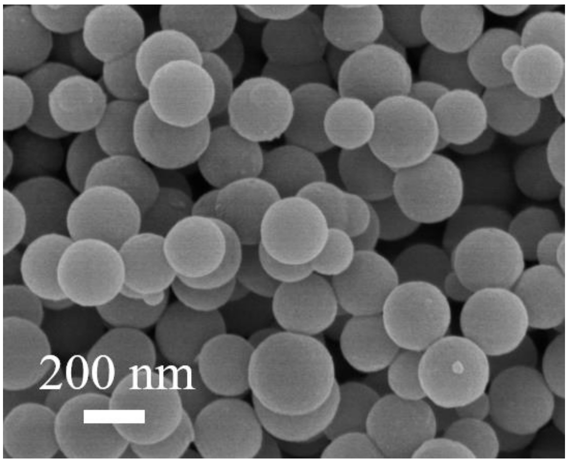

Figure S6. SEM image of pure ACC generated in 5 mM solutions. The average diameter is ~200 nm.

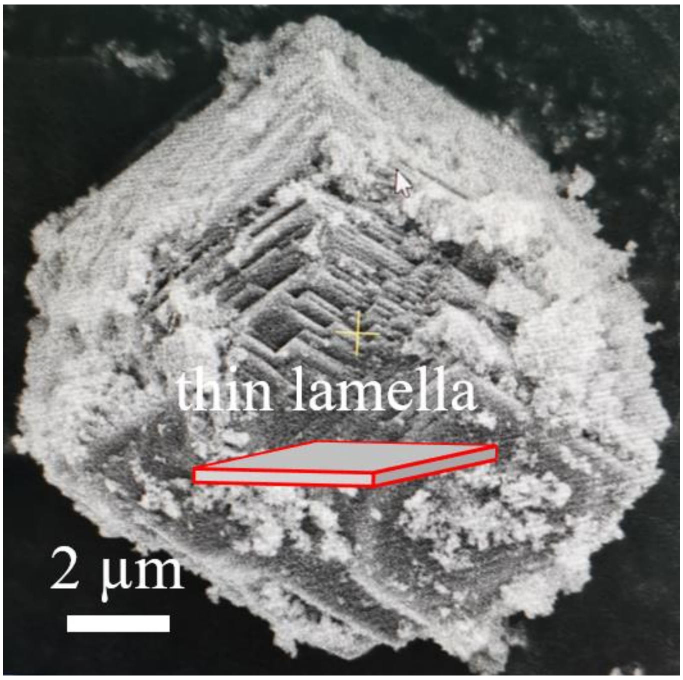

Figure S7. SEM image of the calcite crystal used to prepare the thin lamella (shown in Figure 3a) by focused ion beam (FIB) milling. The position was marked in the image.

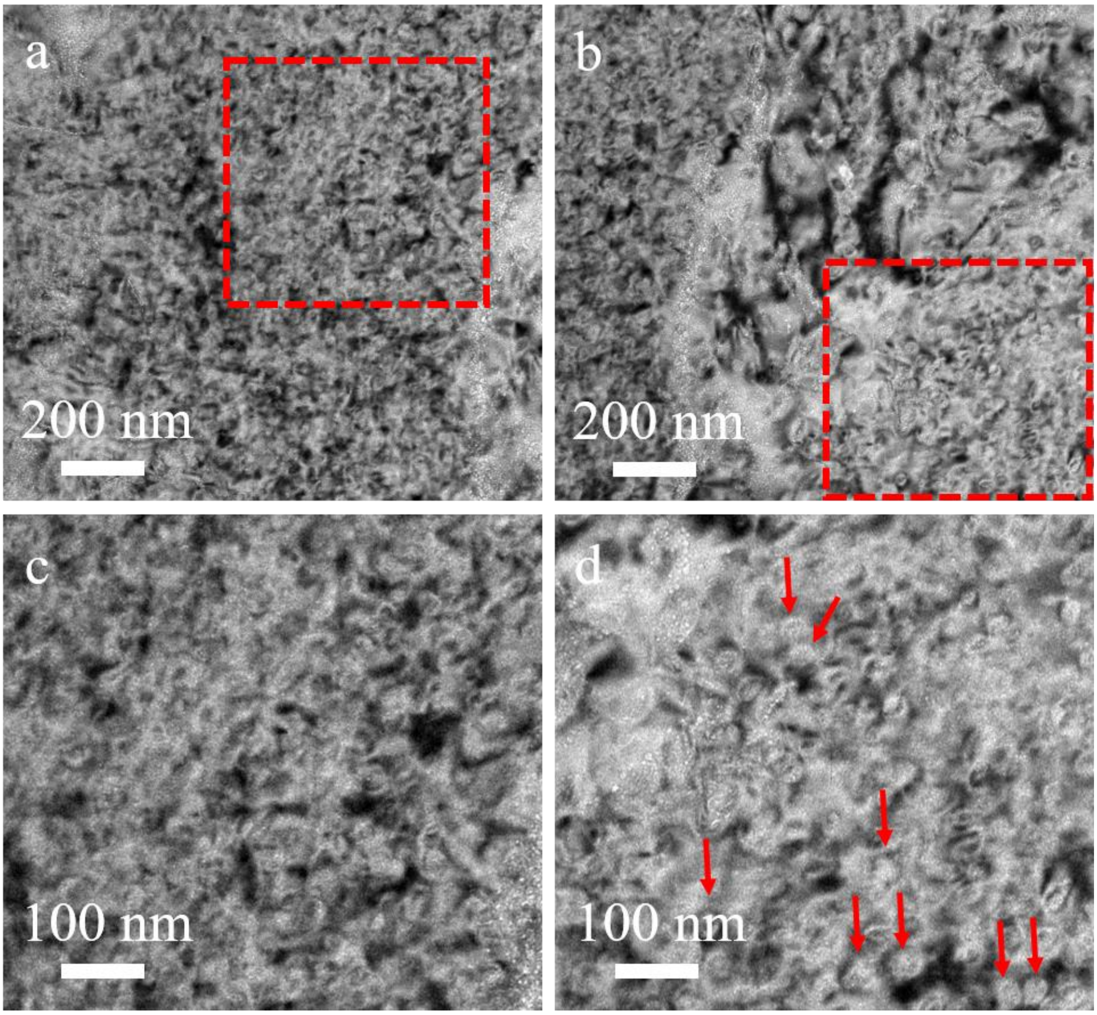

Figure S8. TEM images of the thin lamella of calcite crystal moving from the center outwards the surface. (a) and (b) correspond to regions near the areas 2 and 3 marked in Figure 3a, respectively. (c) and (d) are magnified images of areas marked by rectangle in (a) and (b), respectively. Red arrows in (d) indicate some typical nanoparticles.

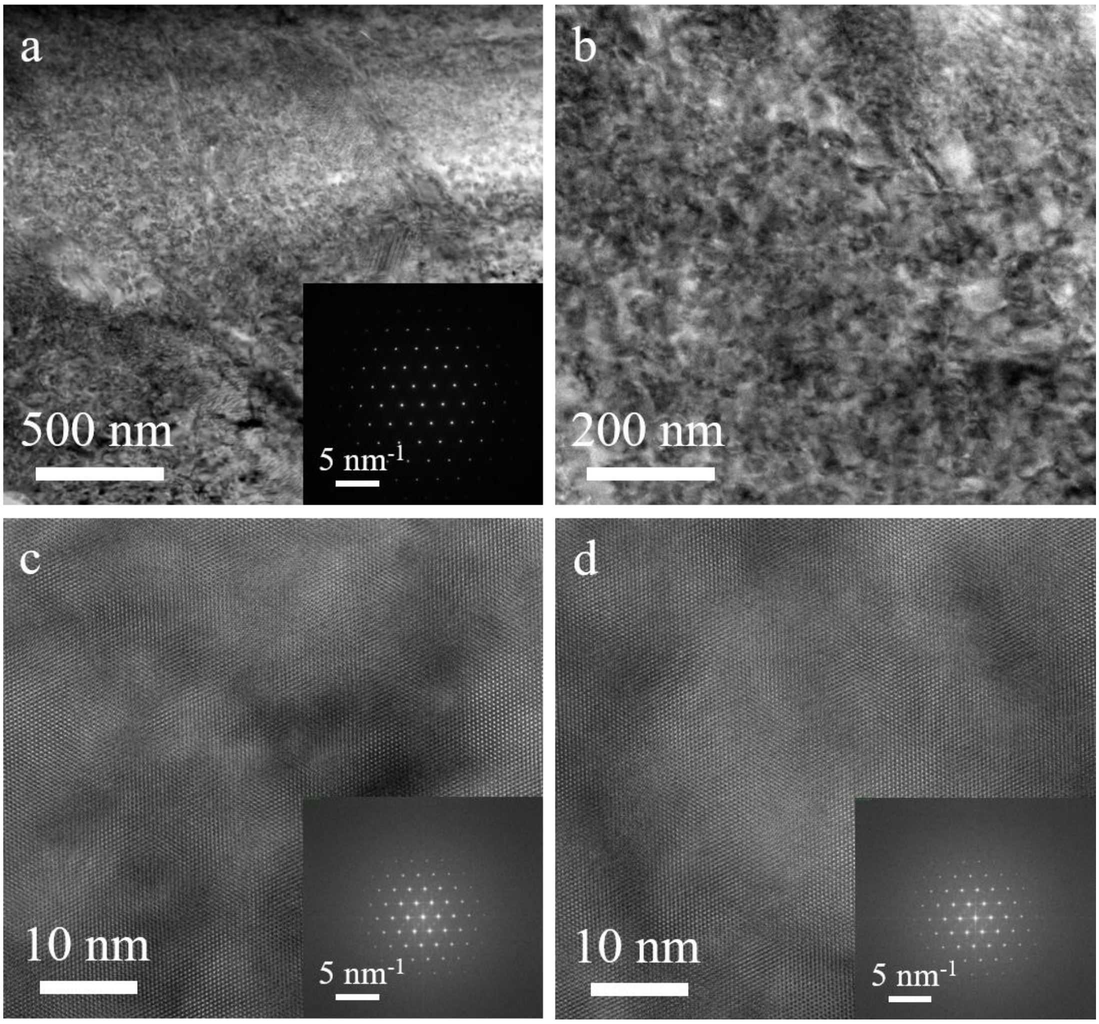

Figure S9. TEM images (a-b) of a thin lamella from another calcite crystal show a similar morphology to the one shown in Figure S8. The inset in (a) is the corresponding electron diffraction pattern, showing the single crystal structure over a large area. High resolution TEM images (c-d) of two regions in (b) show good lattice continuity, and the corresponding FFT images also confirm the single crystal structure.

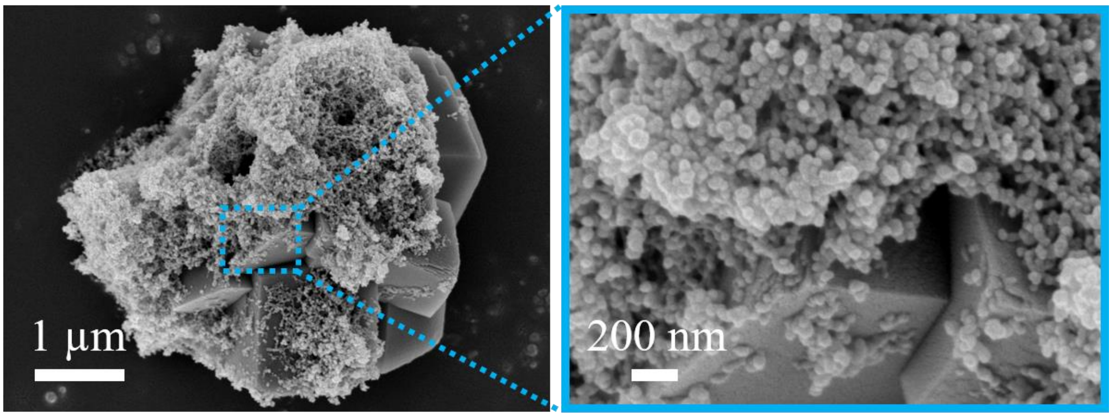

Figure S10. SEM images of precipitate obtained from 2 mM (the concentration of total cationic ions and Na_2CO_3 equals to 2 mM) solution in the presence 5% of Zn^{2+}.
